# Translation, cross-cultural adaptation and validation of the Brazilian version of the Nonarthritic Hip Score

**DOI:** 10.1590/1516-3180.2013.1314487

**Published:** 2013-08-01

**Authors:** Letícia Nunes Carreras Del Castillo, Gustavo Leporace, Themis Moura Cardinot, Roger Abramino Levy, Liszt Palmeira de Oliveira

**Affiliations:** I MSc. Physiotherapist, Department of Surgical Specialties, Faculdade de Ciências Médicas da Universidade do Estado do Rio de Janeiro (FCM-UERJ), Rio de Janeiro, Brazil.; II Physiotherapist, Laboratory of Biomechanics and Motor Behavior, Instituto de Educação Física e do Desporto (IEFD), Universidade do Estado do Rio de Janeiro (UERJ), Rio de Janeiro, Brazil.; III PhD. Associate Professor, Department of Anatomy, Instituto de Biologia (IB), Universidade Federal Rural do Rio de Janeiro (UFRRJ), Seropédica, Brazil.; IV MD, PhD. Associate Professor, Department of Rheumatology, Faculdade de Ciências Médicas da Universidade do Estado do Rio de Janeiro (FCM-UERJ), Rio de Janeiro, Brazil.; V MD, PhD. Associate Professor, Department of Orthopedics, Faculdade de Ciências Médicas da Universidade do Estado do Rio de Janeiro (FCM-UERJ), Rio de Janeiro, Brazil.

**Keywords:** Questionnaires, Quality of life, Translations, Reproducibility of results, Hip, Questionários, Qualidade de vida, Traduções, Reprodutibilidade dos testes, Quadril

## Abstract

**CONTEXT AND OBJECTIVE:**

The Nonarthritic Hip Score (NAHS) is a clinical evaluation questionnaire that was developed in the English language to evaluate hip function in young and physically active patients. The aims of this study were to translate this questionnaire into the Brazilian Portuguese language, to adapt it to Brazilian culture and to validate it.

**DESIGN AND SETTING:**

Cohort study conducted between 2008 and 2010, at Universidade do Estado do Rio de Janeiro (UERJ).

**METHODS:**

Questions about physical activities and household chores were modified to better fit Brazilian culture. Reproducibility, internal consistency and validity (correlations with the Algofunctional Lequesne Index and the Western Ontario and McMaster Universities Arthritis Index [WOMAC]) were tested. The NAHS-Brazil, Lequesne and WOMAC questionnaires were applied to 64 young and physically active patients (mean age, 40.9 years; 31 women).

**RESULTS:**

The intraclass correlation coefficient (which measures reproducibility) was 0.837 (P < 0.001). Bland-Altman plots revealed a mean error in the difference between the two measurements of 0.42. The internal consistency was confirmed through a Cronbach alpha of 0.944. The validity between NAHS-Brazil and Lequesne and between NAHS-Brazil and WOMAC showed high correlations, r = 0.7340 and r = 0.9073, respectively. NAHS-Brazil showed good validity with no floor or ceiling effects.

**CONCLUSION:**

The NAHS was translated into the Brazilian Portuguese language and was cross-culturally adapted to Brazilian culture. It was shown to be a useful tool in clinical practice for assessing the quality of life of young and physically active patients with hip pain.

## RESUMO

**CONTEXTO E OBJETIVO:**

O Nonarthritic Hip Score (NAHS) é um questionário de avaliação clínica que foi desenvolvido na língua inglesa para avaliar a função do quadril em pacientes jovens e fisicamente ativos. O objetivo desse estudo foi traduzir o questionário NAHS para a língua portuguesa do Brasil, adaptá-lo à cultura brasileira e validá-lo.

**TIPO DE ESTUDO E LOCAL:**

Estudo de coorte, realizado entre 2008 e 2010, na Universidade do Estado do Rio de Janeiro (UERJ).

**MÉTODOS:**

Questões sobre atividades físicas e tarefas domésticas foram modificadas para melhor adaptação à cultura brasileira. Reprodutibilidade, consistência interna e validade (correlação entre o Algofunctional Lequesne Index e o Western Ontario McMaster Universities Arthritis Index [WOMAC]) foram testadas. Os questionários NAHS-Brasil, Lequesne e WOMAC foram aplicados em 64 pacientes jovens e fisicamente ativos (média de idade, 40,9 anos; 31 mulheres).

**RESULTADOS:**

O coeficiente de correlação intraclasse que avalia reprodutibilidade mostrou valores de 0,837 (P < 0,001). O gráfico de Bland-Altman revelou um erro médio da diferença das duas medidas de 0,42. A consistência interna foi avaliada pelo alfa de Cronbach com valores de 0,944. A validade entre os questionários NAHS-Brasil/Lequesne e NAHS-Brasil/WOMAC mostraram alta correlação, r = 0,7340 e r = 0,9073; respectivamente. O NAHS-Brasil mostrou boa validade sem efeitos de chão ou de teto.

**CONCLUSÃO:**

O NAHS foi traduzido para a língua portuguesa do Brasil e adaptado para a cultura brasileira, mostrando ser uma ferramenta útil na prática clínica para avaliar a qualidade de vida de pacientes jovens e fisicamente ativos com dor no quadril.

## INTRODUCTION

Studies that evaluate outcomes from clinical or surgical treatments within orthopedics have become more frequent and the emphasis on patients’ opinions has increased.^1^^,^^2^^,^^3^ The most frequently used instruments for hip disease that have been translated into the Portuguese language are the Algofunctional Lequesne Index, Western Ontario and McMasters Universities Arthritis Index (WOMAC) and the Harris Hip Score.^4^^,^^5^^,^^6^ These instruments are used for patients with moderate to severe hip osteoarthritis (OA) or for post-traumatic hip disorders with significant physical limitations. There are questionnaires that evaluate the functional capacity of patients with high physical demands: the Lower Extremity Functional Scale (LEFS), the Modified Harris Hip Score, the Nonarthritic Hip Score (NAHS) and the Hip Outcome Score.^7^^,^^8^^,^^9^^,^^10^ However, with increasing numbers of cases of hip disorders among young and physically active patients, and improvements in diagnostic methods, the need for an instrument that evaluates quality of life in this population has increased.

Our hypothesis was that patients with nearly normal range of motion and pain specifically related to physical activities would require a more specific and sensitive instrument to detect and evaluate functional changes. The NAHS is a simple, short and self-administered questionnaire. It has 20 questions divided into four domains: pain, function, mechanical symptoms and physical activity level.

## OBJECTIVE

The aims of this study were to translate the NAHS through cross-cultural adaptation to Brazilian culture and to test the validity and reproducibility of the Brazilian Portuguese version of the NAHS.

## METHODS

### Type of study

This was a cohort study using data obtained between 2008 and 2010.

### Translation and cross-cultural adaptation procedures

This study was approved by the ethics committee of our Institution and all the subjects provided written informed consent. The translation was approved by Christian P. Christensen, the first author of the NAHS.^9^ To translate and adapt the instrument, the guidelines suggested by Guillemin et al. and revised by Beaton et al. were followed.^11^^,^^12^ The process comprised five steps: translation, back translation, review by a committee, pretesting and final translation.

Two independent translators, who were orthopedic surgeons with experience of hip surgery and were aware of the objectives of the translation, did the initial translation from English into Portuguese. After both initial translations (T1 and T2) had been produced, a combined version (T1,2) was made, based on the two initial translations. This version was then back translated into English by two independent sworn translators (BT1 and BT2) who were not aware of the objective of the translation. A multidisciplinary committee then compared these versions with the original text and a consensus version in Brazilian Portuguese was created (TC1). The option “non-applicable” was added to all questions in the TC1 version.

The TC1 version (pretest) was completed by 10 patients with hip pain and by 20 asymptomatic adults (10 male and 10 female; 10 had had fewer than four years of education and 10 had had postgraduate education). Subjects were excluded from the pretest if they had: (a) visual or cognitive disturbances that did not allow them to read the questionnaire; (b) severe hip limitation characterized by decreased range of motion (less than 10º of internal rotation at 90º hip flexion).

During the translation reviews, questions that had been answered as “non-applicable” were reassessed regarding semantic, idiomatic, cultural and conceptual equivalencies. Conceptual and cultural equivalencies relating to evaluations on physical activities that were not understood by more than 90% of the patients were reevaluated and reworked until they were well understood. During this phase, the TC1 application maintained the conceptual characteristics of the original questionnaire and the objective was to evaluate errors and deviations made during the translation. When there were no more situations that were not part of daily activities, or questions or terms that were not well understood, the TC1 was considered to be the final translation of the questionnaire, without the “non-applicable” option.

### Patients and testing

A consecutive sample of 64 patients with hip injuries and diseases completed the Brazilian Portuguese version of the NAHS and also the Lequesne and WOMAC questionnaires. The patients answered these questionnaires in physicians’ waiting rooms. All the patients completed the study protocol without any loss.

### Reproducibility

We measured the reliability of the questionnaire scores using internal consistency and the test-retest method across repeated administration. To calculate test-retest reliability, all the patients were asked to complete the NAHS-Brazil 48 hours after the first assessment. To minimize the risk of short-term clinical change, no treatment was provided during this period. Agreement was assessed using graphical representations of the measurement error variance between the test and retest answers.

### Validity

Validity is the extent to which a score means what it is supposed to mean, i.e. whether it has the intended interpretation.^13^ In this report, validity was evaluated through two concepts: construct and content. The construct validity of the NAHS-Brazil was evaluated by correlating it with the Brazilian versions of the Lequesne and WOMAC questionnaires. The content validity was tested through the distribution and occurrences of floor and ceiling effects. The floor effect occurs when the minimum possible value is achieved, while the ceiling effect occurs when the maximum possible score is achieved.

### Statistical analysis

Construct validity was tested using Pearson’s correlation coefficient. Internal consistency was calculated using Cronbach’s alpha. This technique was based on the number of items on a scale and the homogeneity of the items. The intraclass correlation coefficient (ICC) was calculated to assess the test-retest reliability. Paired t tests were used to compare and determine statistically significant differences between the first and second assessments. The level of agreement of the test-retest consistency was assessed by plotting Bland-Altman curves. This analysis quantifies agreement through constructing limits of agreement. These statistical limits are calculated by using the mean and standard deviation (SD) of the differences.^14^^,^^15^ The statistical analysis was performed using GraphPad Prism, version 5.00 for Windows (GraphPad Software, USA) and Epi-Info version 3.5.2 (CDC, USA).

## RESULTS

The translation and cross-cultural adaptation was performed based on the original NAHS. The NAHS-Brazil, produced following the cross-cultural adaptation (pretesting), is shown in the appendix.

The Brazilian version of WOMAC was used for the 10 questions that refer to pain and function that originate from WOMAC.^5^ The four questions on mechanical symptoms did not undergo any changes. Four questions on physical activity were modified to better fit Brazilian realities. In the original question on sports with high physical demands, “football” refers to American football, but this was changed to “futebol”, meaning soccer, since this sport is more prevalent in Brazil. Two questions referring to household chores were also modified. In one of the questions, “lifting firewood” was changed to “house cleaning and hand-washing your clothes”, since these are more frequent chores among the Brazilian population. In the other question, “vacuuming and doing laundry” was changed to “doing laundry with a washing machine”. All the changes were approved by the committee and were easily understood in the pretest.

In the final testing, the Brazilian versions of the Lequesne and WOMAC questionnaires were completed concurrently with the NAHS-Brazil by 64 patients with hip complaints. The patients were literate, but their level of schooling was only up to high school level. Some did fitness exercises or hydrotherapy, but none of them were athletes or practiced sports regularly.

Thirty-one patients (48%) were female and 33 (52%) were male. The mean age was 40.9 years (range, 18 to 76 years). The patients had diagnoses of femoroacetabular impingement (24), isolated labral tears (9), pertrochanteric pain syndrome (9), osteonecrosis (6), deep gluteal pain syndrome (5), mild hip osteoarthritis (Tönnis grade 1) (3), rheumatoid arthritis (1), bilateral chondrolysis (1), epiphysiolysis (1), Legg-Perthes disease (1), synovitis (1) and periarticular tendonitis (3) (Table 1).

**Table 1.** Clinical and sociodemographic characteristics of the 64 patients with hip pain

Characteristic	n
Gender	
Female	31
Male	33
Age	
Mean (standard deviation)	40.9 (24.8)
Femoroacetabular impingement	24
Isolated labral tears	9
Pertrochanteric pain syndrome	9
Osteonecrosis	6
Deep gluteal pain syndrome	5
Diagnoses	
Mild hip osteoarthritis (Tönnis 1)	3
Periarticular tendonitis	3
Rheumatoid arthritis	1
Bilateral chondrolysis	1
Epiphysiolysis	1
Legg-Perthes disease	1
Synovitis	1

The mean, standard deviation, minimum and maximum and confidence interval values of each outcome measurement of the Nonarthritic Hip Score (NAHS-Brazil), Lequesne and Western Ontario and McMasters Universities Arthritis Index (WOMAC) questionnaires in the final testing are presented in Table 2.

**Table 2.** Mean values, minimum and maximum values, standard deviation and confidence intervals of the outcome measurements of the Nonarthritic Hip Score (NAHS)-Brazil, Lequesne and Western Ontario and McMasters Universities Arthritis Index (WOMAC) questionnaires

Questionnaires	Mean	Min-Max scores	SD	95% CI
NAHS-Brazil Test	60.7	16.2-96.2	20.7	55.4-65.9
NAHS-Brazil Retest	61.7	8.7-98.7	21.7	56.3-67.1
Lequesne	68.7	16.6-100	18.8	63.9-73.4
WOMAC	66.1	13.5-100	21.2	60.7-71.5

SD = standard deviation; CI = confidence interval; NAHS-Brazil retest was done 48 hours after NAHS-Brazil test

### Test-retest reliability and agreement

**Figure 1.**
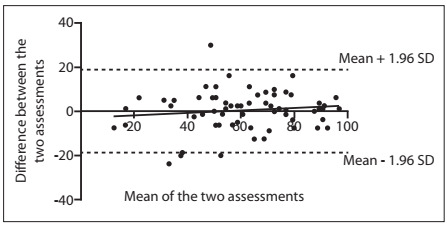
Bland-Altman plot in which the two dotted lines represent limits of agreement (upper and lower). Regression line almost parallel to the X axis demonstrates a fixed bias. SD = standard deviation.

The intraclass correlation coefficient was 0.837 (P < 0.001) and the confidence interval (95% CI) ranged from 0.732 to 0.901. The paired t test did not demonstrate any statistically significant differences between the test-retest means (P = 0.719). Bland-Altman plots revealed a mean error in the difference between the two assessments of 0.42 (SD = 9.21, 95% limit of agreement = -17.62 to 18.48) (Figure 1).

### Internal consistency

Cronbach’s alpha was 0.944 in relation to the NAHS-Brazil total score. Cronbach’s alpha showed values of 0.878, 0.869, 0.920 and 0.901 in relation to the NAHS-Brazil pain, mechanical symptom, function and physical activity domains, respectively. Even with exclusion of each item, the values remained similar to the analysis with all items together (Table 3).

**Table 3.** NAHS-Brazil internal consistency of each domain according to Cronbach’s α values

Domain	Cronbach’s α
Pain	0.878
Mechanical symptoms	0.869
Function	0.920
Physical activity	0.901
Total score	0.944

### Validity

**Figure 2.**
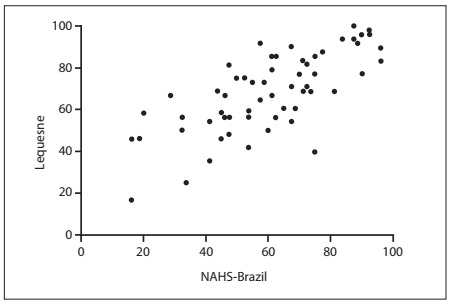
Scatter plot for the Nonarthritic Hip Score (NAHS)-Brazil versus Lequesne.

**Figure 3.**
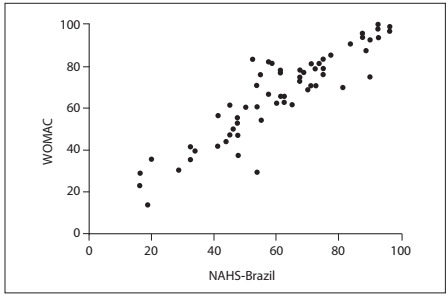
Scatter plot for the Nonarthritic Hip Score (NAHS)-Brazil versus Western Ontario and McMasters Universities Arthritis Index (WOMAC).

**Figure 4.**
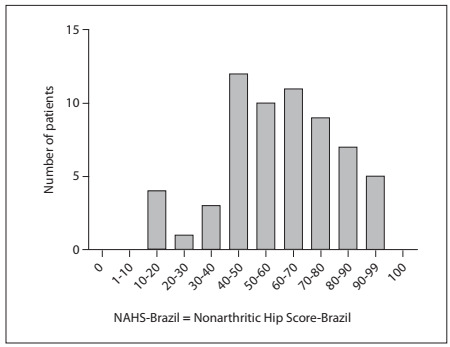
Distribution of patients between ranges of responses.

The NAHS-Brazil showed high correlations with Lequesne (r = 0.7343) and WOMAC (r = 0.9073; P < 0.0001) (Figures 2 and 3). Ceiling effects occurred with the WOMAC and Lequesne questionnaires (Table 2). The NAHS-Brazil showed good content validity; no floor or ceiling effects occurred (Figure 4).

## DISCUSSION

Over the last 50 years, many questionnaires have been developed to evaluate pain and follow up the clinical outcomes of patients with hip OA or subsequent to hip arthroplasty. The most frequently used methods are the Merle D’Aubigné and Postel (MD & Postel), Harris Hip Score (HHS) and WOMAC.^16^^,^^17^^,^^18^ Nonetheless, these questionnaires focus on hip pain and function among elderly people with osteoarthritis.^19^ In our study, we chose to exclude patients with severe functional limitation and those with moderate or severe osteoarthritis.

On this basis, our sample had a low average age (40.9 years old), in relation to other validation studies on questionnaires for hip assessment. In the validation of the Lequesne questionnaire, the patients’ average age was 67 years, and 66% of the patients had moderate or severe osteoarthrosis.^4^ In the validation of the WOMAC questionnaire, the average age was 65 years, and patients with severe osteoarthritis or functional classification IV, according to the criteria of the American College of Rheumatology, were excluded.^5^^,^^20^

Use of these instruments among patients with great range of motion and pain specifically related to physical activities is limited because of the low sensitivity to detection of small, albeit significant, functional changes. With the new diagnostic methods and the new concepts that are applied to hip mechanical alterations in young and physically active patients, questionnaires that evaluate hip OA or hips subsequent to arthroplasty have poor discrimination capabilities with regard to patients who do not have hip OA.^21^

The NAHS is a clinical evaluation questionnaire that was developed to evaluate hip function among young and physically active patients. The NAHS, as well as the majority of the questionnaires evaluating quality of life, was created in the English language. Because of this, these instruments must be translated and cross-culturally adapted in such a way that the semantic, idiomatic, cultural and conceptual equivalencies are maintained.^22^^,^^23^^,^^24^ In this study, some terms were modified, since they did not fit in with Brazilian culture. These were exchanged for other terms that are habitually used in this country, while still maintaining the idea of the effort involved in the activity.

Some authors have taken the view that active participation by the interviewer is required because of the low educational level of the Brazilian population.^25^^,^^26^ However, in translating and validating the NAHS-Brazil, we strictly followed the proposal of the original questionnaire, i.e. a self-administered format, without active participation by the interviewer.

The ICC evaluates the intra and inter-observer reproducibility. It is considered to be excellent when greater than 0.75.^27^ The questionnaire showed excellent reproducibility with ICC values of 0.837, and this is comparable with what was reported for the original version (0.96). However, the values obtained were lower than those found in other validations of hip questionnaires.^4^^,^^5^^,^^10^^,^^28^^,^^29^

The paired t test did not demonstrate any statistically significant differences between the test-retest means. Bland-Altman plots revealed a mean error in the difference between the two assessments of 0.42, with linear regression almost parallel to the axis of the average of the two evaluations, thus indicating that the concordance between the two answers was independent of the patient’s clinical status. This result shows strong evidence that the NAHS-Brazil presents good construct validity, i.e. our results provide evidence that the NAHS-Brazil is a reproducible and valid tool for self-assessment of physical function among young and physically active patients with hip pain, independent of the patient’s functional status. The Cronbach’s alpha values were higher than 0.90 and thus indicate that the NAHS-Brazil has high internal consistency, similar to what has been seen in other studies on translation and validation.^5^^,^^30^^,^^31^

The construct validity was assessed by means of Pearson’s correlation coefficient between NAHS-Brazil and Lequesne and between NAHS-Brazil and WOMAC. We believe that the lower correlation found with the Lequesne questionnaire occurred because this instrument is designed to assess individuals with greater impairment of hip function. The high value for Pearson’s correlation coefficient between NAHS-Brazil and WOMAC demonstrates that these two instruments have similar features; this can be explained by the fact that NAHS includes 10 questions from WOMAC.

The content validity of questionnaires is assessed by means of the distribution and occurrence of floor and ceiling effects.^13^ The NAHS-Brazil showed good content validity; no floor or ceiling effect was observed, i.e. none of the questionnaires presented a score of zero, and none of them showed effects relating to maximum possible score effects. We saw in our study that ceiling effects existed in the Lequesne and WOMAC questionnaires. This result indicates that the content of the NAHS-Brazil seems to be more appropriate for hip evaluations in the population studied.

The NAHS-Brazil is a reliable and validated tool that can be used in clinical practice to evaluate patients with hip pain without osteoarthritis before and after treatment. It can be used with the objective of greater accuracy of discrimination of the clinical hip condition of relatively young patients with little joint degeneration.

The limitations of this study were that the WOMAC questionnaire was used for comparison purposes, given that it shares some questions with the NAHS questionnaire; and that there was no generic questionnaire for comparisons. Another limitation was the need for active participation in questionnaire application by the researcher, among patients with low educational levels. And finally, it has to be considered that the questionnaire should be further evaluated with regard to application among individuals in older age groups than the group studied here.

## CONCLUSION

The Nonarthritic Hip Score was translated into the Brazilian Portuguese language and was cross-culturally adapted to Brazilian culture, following the guidelines for cultural adaptation of quality of life instruments. Its reliability, internal consistency and validity were demonstrated, thus showing that it is a useful tool for assessing the quality of life of young and physically active patients with hip pain, whether at research or at care levels.
